# The association of *Schistosoma* and geohelminth infections with β-cell function and insulin resistance among HIV-infected and HIV-uninfected adults: A cross-sectional study in Tanzania

**DOI:** 10.1371/journal.pone.0262860

**Published:** 2022-01-25

**Authors:** George PrayGod, Suzanne Filteau, Nyagosya Range, Kaushik Ramaiya, Kidola Jeremiah, Andrea M. Rehman, Rikke Krogh-Madsen, Henrik Friis, Daniel Faurholt-Jepsen

**Affiliations:** 1 Mwanza Research Centre, National Institute for Medical Research, Mwanza, Tanzania; 2 Faculty of Epidemiology and Population Health, London School of Hygiene & Tropical Medicine, London, United Kingdom; 3 Muhimbili Research Centre, National Institute for Medical Research, Dar es Saalam, Tanzania; 4 Hindu Mandal Hospital, Dar es Salaam, Tanzania; 5 Centre for Physical Activity Research, Copenhagen University Hospital, Rigshospitalet, Copenhagen, Denmark; 6 Department of Infectious Diseases, Copenhagen University Hospital, Copenhagen, Denmark; 7 Department of Nutrition, Exercise and Sports, University of Copenhagen, Copenhagen, Denmark; 8 Department of Infectious Diseases, Rigshospitalet, Copenhagen, Denmark; Universidad Nacional Autonoma de Mexico, MEXICO

## Abstract

**Objectives:**

Data on the role of helminths on diabetes in Africa are limited. We investigated whether *Schistosoma* and geohelminth infections are associated with β-cell function and insulin resistance among adults.

**Methods:**

A cross-sectional study was conducted among adults during 2016–2017. Demography, *Schistosoma* and geohelminth infections, HIV and insulin data were collected. Insulin during an oral glucose tolerance test (fasting, 30, and 120-min), overall insulin secretion index, insulinogenic index, HOMA-β, and HOMA-IR were main outcome measures for β-cell function and insulin resistance, respectively. Generalized estimating equations and generalized linear models assessed the association of *Schistosoma* and geohelminth infections with outcome measures separately by HIV status. Outcomes were presented as marginal means with 95% CI.

**Results:**

Data were obtained for 1718 participants. *Schistosoma* infection was associated with higher 30-min insulin (24.2 mU/L, 95% CI: 6.9, 41.6) and overall insulin secretion index (13.3 pmol/L/mmol/L; 3.7, 22.9) among HIV-uninfected participants but with lower fasting insulin (-0.9 mU/L; -1.6, -0.2), 120-min insulin (-12.0 mU/L; -18.9, -5.1), and HOMA-IR (-0.3 mmol/L; -0.6, -0.05) among HIV-infected participants not yet on antiretroviral therapy (ART). Among HIV-infected participants not on ART, geohelminth infection was associated with lower fasting insulin (-0.9 mU/L; -1.6, -0.2), 120-min insulin (-9.1 mU/L; -17.3, -1.0), HOMA-β (-8.9 mU/L)/(mmol/L; -15.3, -2.6) and overall insulin release index (-5.1 pmol/L/mmol/L; -10.3, 0.02), although this was marginally significant. There was no association among those on ART.

**Conclusions:**

*Schistosoma* infection was associated with higher β-cell function among HIV-uninfected participants whereas *Schistosoma* and geohelminth infections were associated with reduced β-cell function among HIV-infected participants not on ART.

## Introduction

Schistosomes and geohelminths are neglected human infections with significant morbidity particularly in the global south including Sub-Saharan Africa (SSA) [1]. Besides causing infectious-related ill health, studies have suggested that *Schistosoma* and geohelminth infections may have a protective role on the low-grade chronic inflammation-linked cardiometabolic diseases (including diabetes) [2, 3] as they change Th1 to Th2 immune response i.e. shifting pro-inflammatory to regulatory response [4, 5]. These helminths infections may increase circulating levels of interleukin (IL)-4, IL-5, IL-10 and IL-13 which may act to blunt or reverse the Th1-induced inflammation in metabolic tissues resulting in increased insulin sensitivity [6]. A recent systematic review of four Asian studies showed that helminth infections were associated with lower risk of type 2 diabetes [2]. However, there has been limited work to investigate the role of helminths on diabetes in SSA, where due to significant overlap with other infections like HIV, the association of helminths with diabetes could be different.

Data suggest that HIV infection, in contrast to helminths may increase the risk of diabetes [7, 8]. Epidemiological data report elevated serum levels of inflammatory markers (e.g. IL-6) and C-reactive protein (CRP) and link these to excess risk of diabetes and cardiovascular events [9, 10] suggesting that that inflammatory process and immune-modulation may explain the higher risk. Observational and experimental studies have suggested that schistosomiasis and other helminths could lead to more severe HIV infection characterized by a higher viral load [11–14]. Mice studies link this higher viral load to weakened ability of the host Th1 cellular components to fight viruses and reactivation of latent viruses [15, 16]. We suggest that the immunological shift from Th1 to Th2 exerted by *Schistosoma* and geohelminth infections may suppress Th1 cellular ability to fight HIV virus and bacterial infections leading to significant activation of cellular components of the immune system and chronic inflammation. In this paper we assessed whether people with *Schistosoma* or geohelminth-HIV co-infections compared to those with HIV infection only have more insulin resistance and consequently reduced β-cell function [17]. Also, we assessed if *Schistosoma* and geohelminth infections reduce insulin resistance and as a result enhance β-cell function among HIV-uninfected individuals.

## Materials and methods

### Study design and settings

This was a cross-sectional analysis of baseline data from the Chronic Infections, Co-morbidities and Diabetes in Africa (CICADA) study, a cohort study investigating risk factors for diabetes among HIV-uninfected and HIV-infected adults in north-western Tanzania and registered at clinical.trials.gov as NCT03106480. During October 2016 to November 2017, CICADA recruited 1947 participants and those with both helminth (*Schistosoma* and geohelminth) and insulin data were eligible for inclusion in the current analysis.

### Recruitment of participants

The study population and main methods have been reported recently [7]. Briefly, all surviving HIV-infected individuals from the Nutritional Support for African Adults Starting Antiretroviral Therapy (NUSTART) trial [18] and both HIV-infected and uninfected individuals from TB-NUT (Nutrition, Diabetes and Pulmonary Tuberculosis) study [19, 20] were invited for enrolment in CICADA study. HIV-infected participants from those studies had been on ART a median of 53 months (interquartile range 46; 102 months). In addition, a new cohort of HIV-infected people who visited antiretroviral therapy (ART) clinics in Mwanza City from October 2016 to November 2017, who were preparing to start ART and were not part of TB-NUT or NUSTART cohorts were also invited if they were aged ≥18 years and residents of Mwanza City. Using a computer-generated randomization list, we randomly selected half of the new HIV-infected participants and selected HIV-uninfected participants for frequency matching. Criteria for HIV-negative participants were: lived within the same neighbourhood as the HIV index participant (defined as living in the same street or sub-village), HIV-negative based on HIV rapid tests, had lived in Mwanza City for at least 3 months with no plans for relocating within the next 3 years, aged 18 years or above and age difference from the HIV-infected index participant not more than 5 years, same sex as the HIV-infected index participant, and willing to consent.

### Data collection

#### Demography, socioeconomic status (SES) and NCDs risk factors

Data on demography, level of education, occupation, religion, marital status, alcohol intake, and smoking were collected based on WHO STEPS manual questionnaire [21]. In addition, information on possession of house (including type of toilet used and cooking place), bicycle, motorcycle, vehicle, sewing machine, radio, television, gas cooker, air-condition, mobile phone, animals, chicken, and boat were collected and used to compute SES using principal component analysis [22]. In this paper, SES was divided in tertiles (i.e. lower, middle and upper). Smoking history was classified as never, past and current smoking while alcohol intake was classified to never or ever groups. Reported physical activity was collected using global physical activity questionnaire and computed as metabolic equivalent of tasks minutes per week [23]. Participants were asked for a history of tuberculosis (TB) treatment and being on TB treatment was considered as ongoing clinical TB.

#### Anthropometry and body composition

While barefoot and with minimal clothing, weight of the participant was determined to the nearest 0.1 kg using a digital scale (Seca, Germany), height measured to the nearest 0.1 cm using a stadiometer fixed to the wall (Seca, Germany) and waist circumference using non-stretchable tape to the nearest 0.1 cm. Anthropometric measurements were taken in triplicate and medians were used for analysis. Based on weight and height measurements, body mass index (BMI) was calculated as mass (kg)/height (m)^2^. Fat mass (kg) was determined using a bio-impedance analyser (Tanita BC418, Tokyo, Japan).

#### Glucose, insulin, CRP and HIV assessment

Following 8 hours of overnight fasting, plasma glucose (Hemocue201 RT, Hemocue AB, Angelholm, Sweden) and glycated haemoglobin A1c (HbA1c) (Hemocue HbA1c 501, Hemocue AB, Angelholm, Sweden) were determined using venous blood. Then participants underwent an oral glucose tolerance test (OGTT) with blood collection at 30 and 120 minutes (min). Venous blood samples drawn at the same time as those for glucose assessment were separated into serum for insulin (fasting, 30 min and 120 min) and CRP assessments and stored at -80°C pending analysis. ELISA technique was used to assess insulin in Denmark using dual-monoclonal antibodies (ALPCO, Salem, NH, USA) whereas CRP was measured using sandwich ELISA in Germany [24]. HIV testing was done using two rapid antibody tests (SD HIV- 1/2 3.0 SD standard diagnostics Inc, and The Uni-Gold, Trinity Biotech, IDA Business Park, Bray, Co. Wicklow, Ireland). Discordant samples were tested using Uniform II vironostika-HIV Ag/Ab Micro-Elisa system (Biomerieuxbv, The Netherlands).

#### Parasites assessment

Helminth prevalence was determined using stool and urine samples. Stool was collected for determination of geohelminths (i.e Hookworms, *Ascaris lumbricoides*, *Trichiuris trichiura*, and *Strongyloides stercoralis*) and *Schistosoma (S*.*) mansoni*. Then duplicate smears (41.7 mg) were prepared from each stool sample and examined within 30 minutes of collection by two technicians using the Kato-Katz method [25]; differences in results read by the two technicians were resolved by consensus. Urine samples were examined for *Schistosoma (S*.*) haematobium* eggs in 10 ml of urine according to the nucleopore filtration method. Based on these data, participants with any geohelminth infection were classified as geohelminth-infected whereas those with any *Schistosoma* infection were classified as *Schistosoma*-infected. Also, there were 15 participants (<1%) who had *Schistosoma* and geohelminth co-infection and these were classified as *Schistosoma*-infected. *Schistosoma* and geohelminth groups were the main exposure variables. These were analysed separately because schistosomes are blood-flukes and may lead to more severe tissue pathologies [26] and therefore might have distinct immune modulation characteristics in relation to β-cell function and insulin resistance compared to geohelminth infections. Also using blood sample we determined malaria infection using standard malaria microscopy techniques [27].

#### β-cell function and insulin resistance

Fasting, 30,and 120 min insulin levels, Homeostatic model assessment (HOMA)-β, insulinogenic index and total insulin release index were used as surrogates of β-cell function [28–30] whereas HOMA-Insulin Resistance (IR) was used as surrogate of insulin resistance [30] (S1 Table). In addition, fasting, 30, and 120 min glucose, HbA1c, fat mass and waist circumference were included as secondary markers of β-cell function and insulin resistance. All markers were used as outcome measures.

### Ethics

Ethical clearance was provided by the National Institute for Medical Research (NIMR) in Tanzania and the London School of Hygiene and Tropical Medicine in UK and a consultative approval was obtained from the National Committee on Health Research Ethics in Denmark. Participants were enrolled after completing a written informed consent and those with diabetes and other illnesses were referred to Sekou-Toure referral hospital for care. All data were fully anonymized.

### Data management and statistics

Data were double entered in CSPro database and analysed in STATA version 13 (Station College, Texas, USA). Demographic characteristics, body composition, physical activity, smoking, alcohol drinking, CRP and HIV were presented as percentages, means, and geometric means. Comparison of these variables between participants without helminth infection vs those with *Schistosoma* or geohelminth infection were done using t-test (if variables were continuous or after log-transformation where data were presented as geometric means) and chi-squared test (if variables were categorical).

We investigated the role of *Schistosoma* and geohelminth infections on fasting insulin, 30, and 120 min insulin using generalized estimating equations (GEE) with gamma distribution and log link since data were skewed positively with an unstructured covariance matrix (to account for within-person correlations of these markers at the 3 time points) and robust standard errors. However, for correlated outcomes with normal distribution (fasting glucose, 30, and 120 min glucose) we applied GEE with Gaussian distribution and identity link. For derived markers of β-cell function (i.e. HOMA-β, insulinogenic index and overall insulin release index) and HOMA-IR, which were all positive skewed, we used generalized linear models with gamma distribution and log link to investigate the association of main exposures on these outcomes. Finally, linear regression with robust standard errors was used on to investigate the associations between exposures with HbA1c, fat mass and waist circumference. Analyses were initially adjusted for age and sex and in final models further adjustments for body mass index, physical activity, and CRP were done [31–33] as well as malaria infection and clinical TB co-morbidities which may cofound the hypothesized relationships [34, 35] However, in final models for fat mass and waist circumference we adjusted for alcohol intake [36] and smoking [37, 38] in addition to CRP and physical activity because these could be important confounders. Data on these associations were presented as marginal means with 95% confidence intervals. In all analyses a significance level of *P*<0.05 was used. Effect modification by HIV status was explored by fitting interaction terms where wald tests with *p*<0.05 indicated significant interactions existed.

## Results

**Fig 1:**
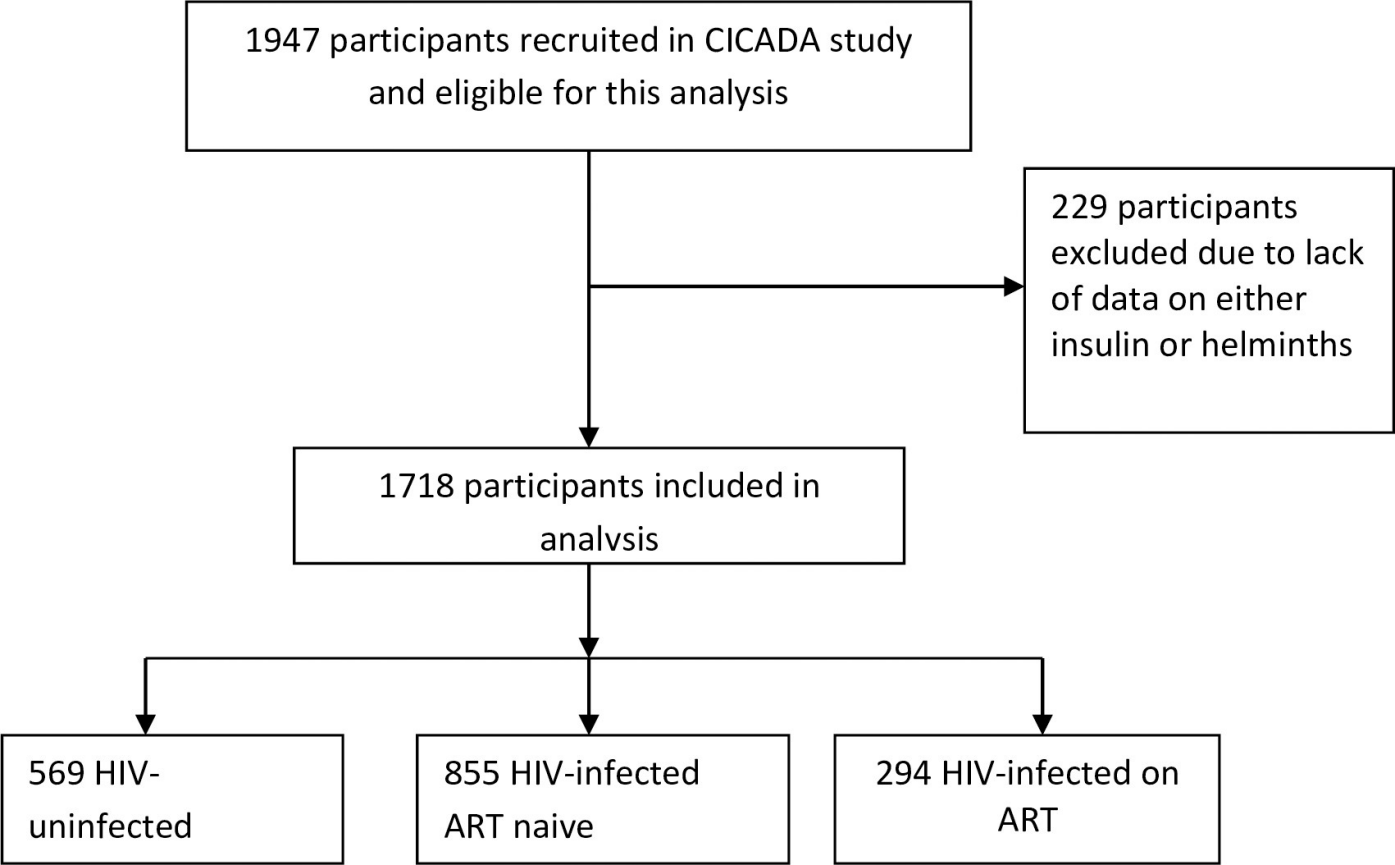
Study flow diagram. ART, Antiretroviral therapy; CICADA, the Chronic Infections, Co-morbidities and Diabetes in Africa.

*Schistosoma*, geohelminth, and insulin data were obtained for 1718 participants (569 HIV-uninfected, 855 HIV infected not on ART and 294 HIV infected on ART) (Fig 1). Background characteristics of participants not included and those included were similar except the proportion of females was higher in those not included (S2 Table). Due to significant interactions between *Schistosoma* or geohelminth infection with HIV treatment status on some outcomes (S3−S6 Tables) data are presented by HIV treatment status. The prevalence of *Schistosoma* infection was 8.9% (51/569), 8.1% (70/855), 6.1% (18/294) and that for geohelminth infection was 8.4% (48/569), 6.7% (57/855) and 10.5% (31/294) among HIV-uninfected, HIV-infected not yet on ART and HIV-infected on ART groups, respectively. Prevalence of individual helminths are presented in (S7 and S8 Tables). Similar to our earlier report [7], the prevalence of diabetes was 4.4% (25/569) among HIV-uninfected, 9.1% (78/855) among HIV-infected not yet on ART, and 3.1% (9/294) among those HIV-infected on ART. In this cohort published CD4 data showed that participants with HIV-infection had lower CD4 count and those on ART seemed to have higher CD4 count compared to those not yet on ART, but lower CD4 count compared to HIV-uninfected participants [7]. Among HIV-uninfected participants, those who were *Schistosoma*-infected were younger compared to helminth uninfected participants (35.9 years vs 43.2 years) and the proportion of women was lower in *Schistosoma*-infected compared to helminth-uninfected participants (Table 1). Similarly, among HIV-uninfected participants, those infected with *Schistosoma* or geohelminths had lower BMI than those not infected (*P*<0.05). However, the level of physical activity was higher in the geohelminth-infected than helminth uninfected participants (*P* = 0.02). Among HIV-infected participants on ART, *Schistosoma* infected participants seemed to have lower prevalence of current smokers compared to geohelminth uninfected group. We found no other major differences within HIV treatment groups.

**Table 1:** Background characteristics by helminth infection status.

	Helminth un-infected	*Schistosoma*-infected^a^	Geohelminth-infected^b^	*P* ^c^	*P* ^d^
**HIV-uninfected participants**	N = 470	N = 51	N = 48		
Age (years), mean (SD)	43.2 (12.8)	35.9 (11.4)	40.5 (15.3)	<0.0001	0.19
Female sex, n(%)	270 (57.5)	17 (33.3)	25 (52.1)	0.001	0.48
Social economic status, n(%)					
Lower	63 (13.4)	3 (5.9)	6 (12.5)	0.11	0.45
Medium	211 (44.9)	30 (58.8)	26 (54.2)		
Higher	196 (41.7)	18 (35.3)	16 (33.3)		
Body mass index (kg), mean (SD)	23.8 (4.9)	22.5 (3.2)	21.5 (3.3)	0.05	0.001
Physically activity (MET min per week), mean (SD)	8809 (6898)^f^	10233 (8527)	11289 (7774)	0.17	0.02
Smoking status, n(%)					
Never	366 (77.9)	36 (70.6)	34 (70.8)	0.40	0.51
Past	64 (13.6)	8 (15.7)	8 (16.7)		
Current	40 (8.5)	7 (13.7)	6 (12.5)		
Ever taken alcohol, n (%)	322 (68.5)	37 (72.6)	27 (56.3)	0.55	0.08
C-Reactive Protein (mg/L)^e^	1.5 (1.3, 1.6)^f^	1.8 (1.3, 2.7)	1.3 (0.9, 1.7)^f^	0.18	0.39
Tuberculosis treatment, n(%)	0 (0)	0 (0)	0 (0)	-	-
Malaria infection, n(%)	5 (1.1)	1 (1.9)	2 (4.2)	0.46	0.13
**HIV-infected not on antiretroviral therapy participants**	N = 728	N = 70	N = 57		
Age (years), mean (SD)	33.3 (11.0)	38.0 (10.7)	37.3 (10.3)	0.88	0.52
Female sex, n(%)	432 (59.3)	40 (57.1)	35 (61.4)	0.72	0.75
Social economic status, n(%)					
Lower	308 (42.3)	34 (48.5)	27 (47.4)	0.23	0.44
Medium	239 (32.8)	16 (22.9)	14 (24.6)		
Higher	181 (24.9)	20 (28.6)	16 (28.0)		
Body mass index (kg), mean (SD)	21.2 (4.1)	20.6 (3.4)	20.4 (3.9)	0.24	0.17
Physically activity (MET min per week), mean (SD)	8955 (7301)^g^	9525 (7970)^g^	10007 (7256)	0.54	0.30
Smoking status, n(%)					
Never	552 (76.0)^g^	43 (63.2)^g^	51 (89.5)	0.02	0.06
Past	92 (12.7)	10 (14.7)	2 (3.5)		
Current	82 (11.3)	15 (22.1)	4 (7.0)		
Ever taken alcohol, n (%)	540 (74.4)^g^	57 (83.8)^g^	19 (33.3)	0.09	0.20
C-Reactive Protein (mg/L)^e^	4.5 (3.9, 5.1)^i^	5.2 (3.3, 8.0)	4.5 (2.9, 7.0)^g^	0.54	0.98
Tuberculosis treatment, n(%)	10 (1.4)	0 (0)	3 (5.3)	1.00	0.06
Malaria infection, n(%)	12(1.7)	2 (2.9)	1 (1.8)	0.35	1.00
**HIV-infected on antiretroviral therapy participants**	N = 245	N = 18	N = 31		
Age (years), mean (SD)	45.8 (9.8)	43.6 (11.2)	41.4 (9.3)	0.37	0.02
Female sex, n(%)	149 (60.8)	12 (66.7)	18 (58.1)	0.62	0.77
Social economic status, n(%)					
Lower	116 (47.4)	5 (27.8)	12 (38.7)	0.27	0.65
Medium	32 (13.0)	3 (16.7)	5 (16.1)		
Higher	97 (39.6)	10 (55.5)	14 (45.2)		
Body mass index (kg), mean (SD)	20.8 (3.8)	19.6 (2.7)	20.4 (3.5)	0.19	0.56
Physically activity (MET min per week), mean (SD)	8694 (7360)	10789 (7806)	11652 (8455)	0.25	0.04
HIV patients on protease inhibitors, n(%)	14 (5.7)	0 (0)	2 (6.5)	0.61	0.70
Smoking status, n(%)					
Never	173 (70.6)	12 (66.7)	24 (77.4)	0.75	0.88
Past	60 (24.5)	5 (27.8)	6 (19.4)		
Current	12 (4.9)	1(5.5)	1 (3.2)		
Ever taken alcohol, n (%)	182 (74.3)	12 (66.7)	20 (64.5)	0.48	0.25
C-Reactive Protein (mg/L)^e^	2.4 (2.0, 2.8)^h^	3.0 (1.3, 6.9)	1.9 (1.1, 3.2)^g^	0.45	0.36
Tuberculosis treatment, n(%)	2 (0.82)	0 (0)	1 (3.2)	1.00	0.30
Malaria infection, n(%)	9 (3.7)	1 (5.6)	3 (9.7)	0.51	0.14

SD, Standard deviation; MET, Metabolic equivalent of task

^a^Included both *Schistosoma mansoni* and *Schistosoma haematobium*

^b^Included hookworms, *Ascaris lumbricoides*, *Strongyloides stercoralis* and *Trichuris trichiura*

^c^Difference between helminth un-infected and *Schistosoma* infected groups

^d^Difference between helminth un-infected and geohelminth infected groups

^e^Data presented as geometric mean (95%CI)

^f^1participant missing

^g^2 participants missing

^h^3 participants missing

^i^5participants missing

### Association of *Schistosoma* infection with β-cell function and insulin resistance

Table 2 presents associations of *Schistosoma* infection with markers of β-cell function and insulin resistance. Among the HIV-uninfected group, in final models, *Schistosoma* infection was associated with higher 30 min insulin (24.2 mU/L, 95% CI: 6.9, 41.6) and overall insulin release index (13.3 pmol/L/mmol/L, 95%CI: 3.7, 22.9) but there was no association with HOMA-IR. In contrast among the HIV-infected not yet on ART group, *Schistosoma* infection was associated with lower fasting insulin (-0.9 mU/L, 95%CI: -1.6, -0.2) and 120 min insulin (-12.0 (mU/L, 95% CI: 18.9, -5.1) as well as HOMA-IR (-0.3 mmol/L, 95%CI: -0.6, -0.05). Among HIV-infected participants on ART 30 min insulin, 120 min insulin, HOMA-β, insulinogenic index and overall insulin release index tended to be lower in participants with *Schistosoma* infection, but no differences were statistically significant.

**Table 2:** Analysis of association of *Schistosoma* infection with β-cell function and insulin resistance by HIV treatment status.

	Age and sex adjusted model				Fully adjusted model^a^			
	Marginal means (95% CI)			*P*	Marginal means (95% CI)			*P*
	*Schistosoma*-uninfected	*Schistosoma*-infected	Difference		*Schistosoma*-uninfected	*Schistosoma* infected	Difference	
HIV-negative participants (N = 569)								
Insulin level during OGTT								
Fasting insulin (mU/L)	7.2 (6.7, 7.8)	6.9 (5.9, 7.9)	-0.3 (-1.4, 0.8)	0.57	7.0 (6.5, 7.5)	7.2 (6.3, 8.2)	0.2 (-0.8, 1.3)	0.69
Insulin at 30 min (mU/L)	54.3 (50.4, 58.1)	74.6 (58.9, 90.3)	20.3 (4.1, 36.6)	0.01	54.6 (51.1, 58.1)	78.8 (62.0, 95.9)	24.2 (6.9, 41.6)	0.006
Insulin at 120 min (mU/L)	49.3 (46.1, 52.6)	51.6 (38.9, 64.3)	2.2 (-10.9, 15.4)	0.73	50.1 (47.0, 53.2)	51.6 (41.3, 62.0)	1.5 (-9.2, 12.2)	0.78
Markers of β-cell function								
HOMA-β (mU/L)/(mmol/L)	51.8 (47.5, 56.2)	52.5 (38.9, 65.9)	0.6 (-13.6, 14.8)	0.93	51.5 (47.4, 55.6)	56.3 (43.0, 69.5)	4.7 (-9.0, 18.5)	0.50
Insulinogenic index (mU/L)/(mg/dL)	1.8 (1.5, 2.2)	3.3 (1.2, 5.5)	1.5 (-0.7, 3.6)	0.18	1.9 (1.5, 2.3)	3.1 (1.3, 4.8)	1.2 (-0.6, 2.9)	0.19
Overall insulin release index (pmol/L/mmol/L)	41.5 (39.0, 43.9)	51.7 (42.0, 61.4)	10.2 (0.2, 20.3)	0.04	41.2 (38.9, 43.5)	54.5 (45.1, 64.0)	13.3 (3.7, 22.9)	0.01
Marker of insulin resistance								
HOMA-IR (mU/L)/(mmol/L)	2.2 (1.9, 2.3)	2.1 (1.5, 2.6)	-0.1 (-0.6, 0.5)	0.76	2.1 (1.9, 2.3)	2.2 (1.7, 2.6)	0.05 (-0.4, 0.5)	0.86
HIV-infected not on antiretroviral therapy (N = 855)								
Insulin level during OGTT								
Fasting insulin (mU/L)	6.0 (5.7, 6.4)	4.9 (4.2, 5.7)	-1.1 (-1.9,-0.3)	0.01	5.9 (5.6, 6.2)	5.0 (4.4, 5.7)	-0.9 (-1.6, -0.2)	0.01
Insulin at 30 min (mU/L)	49.4 (46.5, 52.2)	46.3 (38.6, 54.1)	-3.1 (-11.8, 5.2)	0.48	49.5 (46.9, 52.1)	48.8 (40.8, 56.9)	-0.7 (-9.0, 7.7)	0.87
Insulin at 120 min (mU/L)	49.3 (46.3, 52.3)	37.1 (29.4, 44.8)	-12.2 (-20.4,-4.0)	0.01	49.8 (46.9, 52.6)	37.8 (31.4, 44.2)	-12.0 (-18.9, -5.1)	0.001
Markers of β-cell function								
HOMA-β (mU/L)/(mmol/L)	43.2 (40.7, 45.7)	38.4 (31.3, 45.5)	-4.8 (-12.3, 2.7)	0.21	43.1 (40.8, 45.5)	41.1 (34.3, 48.0)	-2.0 (-9.1, 5.1)	0.58
Insulinogenic index (mU/L)/(mg/dL)	1.4 (1.2, 1.6)	2.0 (1.1, 2.9)	0.6 (-0.3, 1.6)	0.19	1.4 (1.2, 1.6)	2.1 (1.1, 3.1)	0.7 (-0.3, 1.7)	0.18
Overall insulin release index (pmol/L/mmol/L)	37.5 (35.6, 39.3)	32.7 (27.6, 37.9)	-4.7 (-10.2, 0.7)	0.09	37.3 (35.7, 39.0)	33.8 (29.1, 38.5)	-3.5 (-8.5, 1.4)	0.16
Marker of insulin resistance								
HOMA-IR (mU/L)/(mmol/L)	1.8 (1.7, 1.9)	1.4 (1.1, 1.7)	-0.4(-0.7, -0.04)	0.02	1.8 (1.7, 1.9)	1.5 (1.2, 1.7)	-0.3 (-0.6, -0.05)	0.02
HIV infected on antiretroviral therapy (N = 294)								
Insulin level during OGTT								
Fasting insulin (mU/L)	6.5 (5.9, 7.1)	6.2 (4.8, 7.5)	-0.3 (-1.9, 1.1)	0.62	6.5 (6.0, 7.0)	6.8 (5.0, 8.5)	0.3 (-1.5, 2.1)	0.76
Insulin at 30 min (mU/L)	48.4 (43.3, 53.6)	40.6 (27.3, 53.9)	-7.8 (-22.0, 6.5)	0.28	48.5 (43.8, 53.2)	41.4 (31.3, 51.5)	-7.1 (-17.8, 3.6)	0.19
Insulin at 120 min (mU/L)	36.5 (33.2, 39.9)	32.7 (22.8, 42.7)	-3.8(-14.3, 6.6)	0.49	37.2 (33.8, 40.6)	33.5 (25.4, 41.6)	-3.7 (-12.1, 5.0)	0.41
Markers of β-cell function								
HOMA-β (mU/L)/(mmol/L)	44.1 (39.7, 48.5)	37.8 (23.9, 51.6)	-6.3 (-20.8, 8.2)	0.39	44.2 (39.9, 48.5)	42.8 (28.3, 57.2)	-1.4 (-16.3, 13.4)	0.85
Insulinogenic index (mU/L)/(mg/dL)	1.3 (0.9, 1.6)	0.8 (-0.1, 1.7)	-0.5 (-1.5, 0.5)	0.34	1.3 (0.8, 1.8)	0.7 (-0.3, 1.7)	-0.6 (-1.8, 0.6)	0.29
Overall insulin release index (pmol/L/mmol/L)	33.2 (30.4, 36.0)	28.4 (19.6, 37.2)	-4.9 (-14.1, 4.4)	0.30	33.4 (30.7, 36.1)	28.2 (19.8, 36.2)	-5.2 (-13.7, 3.2)	0.22
Marker of insulin resistance								
HOMA-IR (mU/L)/(mmol/L)	2.0 (1.8, 2.2)	1.8 (1.2, 2.5)	-0.2 (-0.8, 0.5)	0.65	1.9 (1.8, 2.1)	2.1 (1.5, 2.8)	0.15 (-0.5, 0.8)	0.64

HOMA-β, Homeostatic model assessment-β; HOMA-IR, HOMA-Insulin Resistance; OGTT, oral glucose tolerance test.

^a^Adjusted for age, sex, C-Reactive Protein, body mass index, malaria infection, tuberculosis and physical activity.

### Association of geohelminth infection with β-cell function and insulin resistance

Table 3 presents the association of geohelminth infection with markers of β-cell function and insulin resistance. In final adjusted models, we found that among the HIV-uninfected group, geohelminth infection was associated with lower insulinogenic index (-0.9(mU/L)/(mg/dL, 95% CI:—1.7, -0.1), but there was no association with insulin resistance. In addition, among HIV-infected participants not yet on ART, geohelminth infection was associated with lower fasting insulin (-0.9 mU/L, 95% CI: -1.6, -0.2), 120 min insulin (-9.1 mU/L, 95%CI: -17.3, -1.0), HOMA-β (-8.9 mU/L)/(mmol/L, 95% CI: -15.3, -2.6), and overall insulin release index (-5.1 pmol/L/mmol/L, 95%CI: -10.3, 0.02), although this was marginally significant. Among HIV-infected participants on ART we found no association with geohelminths.

**Table 3:** Analysis of association of geohelminth infection with β-cell function and insulin resistance by HIV treatment status.

	Age and sex adjusted model				Fully adjusted model^a^			
	Marginal means (95% CI)			*P*	Marginal means (95% CI)			*P*
	Geohelminth-uninfected	Geohelminth- infected	Difference		Geohelminth-uninfected	Geohelminth-infected	Difference	
HIV-negative participants (N = 569)								
Insulin level during OGTT								
Fasting insulin (mU/L)	7.2 (6.7, 5.8)	5.7 (4.7, 6.7)	-1.5 (-2.7, -0.4)	0.01	7.0 (6.5, 7.4)	6.6 (5.5, 7.4)	-0.4 (-1.5, 0.7)	0.49
Insulin at 30 min (mU/L)	54.4 (50.6, 58.30	51.7 (39.8, 63.60	-2.7 (-15.2, 9.8)	0.67	54.4 (50.9, 57.9)	59.4 (46.9, 71.9)	5.0 (-7.9, 17.8)	0.45
Insulin at 120 min (mU/L)	49.6 (46.3, 52.8)	46.0 (32.5, 59.5)	-3.6 (-17.5, 10.4)	0.62	50.1 (46.9, 53.2)	50.0 (39.4, 60.5)	-0.1 (-10.9, 10.8)	0.98
Markers of β-cell function								
HOMA-β (mU/L)/(mmol/L)	51.9(47.7, 56.3)	38.9 (28.9, 49.1)	-13.0 (-24.0, -2.0)	0.02	51.2 (47.1, 55.2)	46.5 (35.2, 57.8)	-4.7(-16.5, 7.1)	0.43
Insulinogenic index (mU/L)/(mg/dL)	1.8 (1.3, 2.4)	0.6 (-0.01, 1.2)	-1.3 (-2.0,-0.5)	0.02	1.8 (1.4, 2.2)	0.9 (0.1, 1.7)	-0.9 (-1.7, -0.1)	0.03
Overall insulin release index (pmol/L/mmol/L)	41.6 (39.0, 44.1)	37.9 (30.6, 45.2)	-3.6 (-11.4, 4.1)	0.36	41.1 (38.8, 43.5)	42.0 (34.7, 49.4)	0.9 (-6.7, 8.6)	0.81
Marker of insulin resistance								
HOMA-IR (mU/L)/(mmol/L)	2.2 (1.9, 2.4)	1.8 (1.3, 2.2)	-0.4 (-0.9, 0.1)	0.11	2.1 (1.9, 2.3)	2.1 (1.7, 2.6)	0.02 (-0.5,0.5)	0.92
HIV-infected not on antiretroviral therapy (N = 855)								
Insulin level during OGTT								
Fasting insulin (mU/L)	6.0 (5.7, 6.4)	4.8 (4.1, 5.5)	-1.2 (-1.9, 0.4)	0.02	5.9 (5.6, 6.2)	5.0 (4.4, 5.6)	-0.9 (-1.6, -0.2)	0.01
Insulin at 30 min (mU/L)	49.4 (46.5 52.3)	42.0 (34.7, 49.3)	-7.4 (-15.2, 0.5)	0.06	49.5 (46.9, 52.1)	43.8 (36.5, 51.2)	-5.6 (-13.4, 2.1)	0.15
Insulin at 120 min (mU/L)	49.2 (46.2, 52.1)	36.6 (30.0, 43.1)	-12.6 (-19.8, -5.4)	0.001	49.5 (46.8, 52.3)	40.4 (32.7, 48.1)	-9.1 (-17.3, -1.0)	0.03
Markers of β-cell function								
HOMA-β (mU/L)/(mmol/L)	43.2 (40.7, 45.7)	32.1 (25.6, 38.6)	-11.1 (-18.1, -4.2)	0.002	43.1 (40.8, 45.4)	34.2 (28.1, 40.3)	-8.9 (-15.3, -2.6)	0.01
Insulinogenic index (mU/L)/(mg/dL)	1.4 (1.2, 1.6)	1.9 (1.1, 2.8)	0.6 (-0.3, 1.4)	0.19	1.4 (1.2, 1.6)	2.0 (1.1, 2.9)	0.6 (-0.3, 1.6)	0.20
Overall insulin release index (pmol/L/mmol/L)	37.4 (35.6, 39.3)	30.6 (25.3, 35.9)	-6.8 (-12.4, -1.2)	0.02	37.2 (35.6, 38.9)	32.1 (27.2, 37.1)	-5.1 (-10.3, 0.02)	0.05
Marker of insulin resistance								
HOMA-IR (mU/L)/(mmol/L)	1.8 (1.7, 1.9)	1.5 (1.1, 1.8)	-0.3 (-0.7, 0.01)	0.06	1.8 (1.7, 1.9)	1.6 (1.3, 1.9)	-0.2 (-0.6, 0.1)	0.19
HIV infected on antiretroviral therapy (N = 294)								
Insulin level during OGTT								
Fasting insulin (mU/L)	6.5 (5.9, 7.1)	7.4 (5.6, 9.2)	0.9 (-0.9, 2.8)	0.33	6.5 (6.0, 6.9)	7.4 (6.0, 6.9)	0.9 (-0.5, 2.4)	0.18
Insulin at 30 min (mU/L)	48.3 (43.1, 53.40	59.9 (44.0, 75.7)	11.6 (-5.1, 28.3)	0.17	48.6 (43.9, 53.4)	59.8 (47.2, 72.3)	11.1 (-1.9, 24.2)	0.10
Insulin at 120 min (mU/L)	36.5 (33.1, 39.8)	44.7 (28.8, 60.6)	8.2 (-7.9, 24.4)	0.32	37.4 (33.9, 40.7)	43.5 (32.8, 54.1)	6.1 (-4.9, 17.1)	0.27
Markers of β-cell function								
HOMA-β (mU/L)/(mmol/L)	43.8 (39.4, 48.3)	51.8 (36.8, 66.7)	7.9 (-7.6, 23.5)	0.32	44.5 (40.2, 48.9)	51.4 (38.5, 64.5)	6.9 (-6.6, 20.4)	0.31
Insulinogenic index (mU/L)/(mg/dL)	1.3 (0.9,1.7)	1.4 (0.2, 2.6)	0.1 (-1.1, 1.4)	0.85	1.3 (0.8, 1.8)	1.3 (0.03, 2.7)	0.04 (-1.4,1.4)	0.96
Overall insulin release index (pmol/L/mmol/L)	33.2 (30.3, 36.1)	41.3 (31.1, 51.5)	8.1 (-2.5, 18.7)	0.13	33.5 (30.8, 36.2)	40.9 (32.0, 49.9)	7.4 (-1.8, 16.7)	0.11
Marker of insulin resistance								
HOMA-IR (mU/L)/(mmol/L)	1.9 (1.8, 2.2)	2.2 (1.6, 2.8)	0.2 (-0.4, 0.8)	0.53	1.9 (1.8, 2.2)	2.2 (1.8, 2.7)	0.3 (-0.3, 0.8)	0.32

HOMA-β, Homeostatic model assessment-β; HOMA-IR, HOMA-Insulin Resistance; OGTT, oral glucose tolerance test.

^a^Adjusted for age, sex, C-Reactive Protein, body mass index, malaria infection, tuberculosis and physical activity.

### Association of helminth infections with glucose, HbA1c and body composition

There was no association between *Schistosoma* infection with glucose, HbA1c, fat mass and waist circumference in either HIV-infected group (Table 4). However, among HIV-uninfected participants, geohelminth infection was associated with lower fat mass and waist circumference (*P*<0.005, all) and with HbA1c, although this was only marginally significant (*P* = 0.06) (Table 5)

**Table 4:** Analysis of association of *Schistosoma* infection with glucose, HbA1c, fat mass, and waist circumference by HIV treatment status.

	Age and sex adjusted model				Fully adjusted model^a^^,^ ^b^			
	Marginal means (95% CI)			*P*	Marginal means (95% CI)			*P*
	*Schistosoma*-uninfected	*Schistosoma*- infected	Difference		*Schistosoma*-uninfected	*Schistosoma*-infected	Difference	
HIV-uninfected participants (N = 569)								
Fasting glucose (mmol/L)	6.6 (6.5, 6.7)	6.8 (6.3, 7.3)	0.2 (-0.4, 0.7)	0.56	6.6 (6.5, 6.7)	6.8 (6.3, 7.3)	0.2 (-0.4, 0.7)	0.44
Glucose at 30 min(mmol/L)	8.4 (8.3, 8.6)	8.5 (7.9, 9.2)	0.1 (-0.5, 0.8)	0.73	8.4 (8.3, 8.6)	8.5 (7.9, 9.2)	0.1 (-0.6, 0.8)	0.80
Glucose at 120 min (mmol/L)	8.0 (7.8, 8.3)	8.0 (7.0, 9.1)	0.06 (-0.9, 1.1)	0.91	8.0 (7.8, 8.3)	8.0 (7.8, 8.3)	0.02 (-1.0, 1.1)	0.96
HbA1c (%)	5.5 (5.4, 5.6)	5.7 (5.3, 6.1)	0.2 (-0.3, 0.6)	0.49	5.5 (5.4, 5.6)	5.6 (5.2, 6.1)	0.15 (-0.3, 0.6)	0.45
Fat mass (kg)	16.3 (15.6, 17.1)	15.5 (13.5, 17.5)	-0.8 (-2.9, 1.3)	0.46	14.8 (13.7, 15.9)	13.9 (11.8, 16.0)	-0.9 (-2.9, 1.1)	0.37
Waist circumference (cm)	83.8 (82.7, 84.9)	83.0 (80.4, 85.7)	-0.8 (-3.6, 2.0)	0.57	81.6 (80.1, 83.2)	81.0 (78.1, 83.9)	-0.6 (-3.3, 2.1)	0.66
HIV-infected not on antiretroviral therapy (N = 855)								
Fasting glucose (mmol/L)	6.6 (6.5, 6.6)	6.4 (6.1, 6.6)	-0.2 (-0.5, 0.1)	0.20	6.5 (6.5, 6.6)	6.3 (6.0, 6.6)	-0.2 (-0.5, 0.1)	0.19
Glucose at 30 min(mmol/L)	8.5 (8.4, 8.6)	8.4 (8.0, 8.9)	-0.1 (-0.5, 0.4)	0.80	8.5 (8.4, 8.6)	8.4 (7.9, 8.9)	-0.1 (-0.6, 0.4)	0.68
Glucose at 120 min (mmol/L)	8.5 (8.3, 8.7)	8.3 (7.7, 8.8)	-0.2 (-0.9, 0.3)	0.37	8.5 (8.3, 8.7)	8.2 (7.7, 8.8)	-0.3 (-0.9,0.3)	0.38
HbA1c (%)	5.9 (5.8, 5.9)	5.8 (5.6, 6.0)	-0.1 (-0.3, 0.2)	0.66	5.8 (5.7, 5.9)	5.8 (5.6, 6.0)	-0.04 (-0.2,0.15)	0.68
Fat mass (kg)	11.4 (10.9, 11.9)	10.0 (8.3, 11.6)	-1.4 (-3.2, 03)	0.11	10.5 (9.6, 11.4)	9.3 (7.6, 11.0)	-1.2 (-2.9, -0.5)	0.18
Waist circumference (cm)	77.0 (76.4, 77.7)	76.1 (74.2, 78.1)	-0.9 (-2.9, 1.1)	0.37	76.5 (75.5, 77.6)	75.8 (73.7, 77.8)	-0.7 (-2.7, 1.2)	0.46
HIV infected on antiretroviral therapy (N = 294)								
Fasting glucose (mmol/L)	6.7 (6.6, 6.9)	6.7 (6.5, 7.0)	-0.01 (-0.3, 0.3)	0.94	6.7 (6.6, 6.9)	6.8 (6.5, 7.1)	0.1 (-0.2, 0.4)	0.64
Glucose at 30 min(mmol/L)	8.6 (8.4, 8.8)	8.6 (8.0, 9.1)	-0.03 (-0.6, 0.6)	0.92	8.6 (8.4, 8.8)	8.6 (8.1, 9.2)	0.05 (-0.6, 0.7)	0.88
Glucose at 120 min (mmol/L)	8.0 (7.7, 8.3)	8.3 (7.6, 8.9)	0.3 (-0.5, 1.1)	0.48	8.0 (7.7, 8.2)	8.4 (7.6, 9.1)	0.4 (-0.4, 1.2)	0.33
HbA1c (%)	5.5 (5.4, 5.7)	5.3 (5.0, 5.6)	-0.2 (-0.6, 0.1)	0.20	5.5 (5.3, 5.7)	5.3 (4.9, 5.7)	-0.2 (-0.6, 0.2)	0.27
Fat mass (kg)	11.1 (10.2, 11.9)	11.2 (8.1, 14.3)	0.1 (-3.1, 3.4)	0.94	9.4 (8.1, 10.7)	10.5 (7.3, 13.7)	1.1 (-2.2, 4.4)	0.52
Waist circumference (cm)	78.4 (77.2, 79.6)	76.2 (72.9, 79.5)	-2.2 (-5.7,1.3)	0.21	75.8 (74.1, 77.6)	75.2 (71.9, 78.5)	-0.6 (-4.0, 2.8)	0.72

^a^Adjusted for age, sex, C-Reactive Protein, malaria infection, tuberculosis, body mass index, and physical activity in estimating association with glucose (fasting, 30 and 120 min) and HbA1c

^b^Adjusted for age, sex, C-Reactive Protein, malaria infection, tuberculosis, smoking, alcohol drinking and physical activity in estimating association with fat mass and waist circumference.

**Table 5:** Analysis of association of geohelminth infection with glucose, HbA1c, fat mass, and waist circumference by HIV treatment status.

	Age and sex adjusted model				Fully adjusted model^a^^,^ ^b^			
	Marginal means (95% CI)			*P*	Marginal means (95% CI)			*P*
	geohelminth-uninfected	geohelminth- infected	Difference		geohelminth-uninfected	geohelminth-infected	Difference	
HIV-uninfected participants (N = 569)								
Fasting glucose (mmol/L)	6.6 (6.5, 6.7)	6.7 (6.4, 7.1)	0.1 (-0.3, 0.4)	0.57	6.6 (6.5, 6.7)	6.8 (6.4, 7.2)	0.2 (-0.2, 0.6)	0.27
Glucose at 30 min(mmol/L)	8.5 (8.3, 8.6)	8.5 (8.1, 8.9)	0.04 (-0.4, 0.4)	0.86	8.5 (8.3, 8.6)	8.6 (8.2, 8.9)	0.1 (-0.3, 0.5)	0.55
Glucose at 120 min (mmol/L)	8.0 (7.8, 8.3)	7.8 (7.3, 8.3)	-0.2 (-0.8, 0.3)	0.43	8.0 (7.8, 8.3)	7.9 (7.4, 8.3)	-0.1 (-0.6, 0.4)	0.56
HbA1c (%)	5.5 (5.4, 5.6)	5.2 (5.0, 5.4)	-0.3 (-0.5, -0.1)	0.005	5.5 (5.4, 5.6)	5.3 (5.1, 5.5)	-0.2 (-0.4, -0.01)	0.06
Fat mass (kg)	16.4 (15.6, 17.2)	11.4 (10, 13.0)	-5.0 (-6.9, -3.2)	<0.0001	14.8 (13.7, 16.0)	11.1 (9.3, 12.9)	-3.7 (-5.5, -1.9)	<0.0001
Waist circumference (cm)	83.9 (75.3, 80.2)	77.7 (75.3, 80.2)	-6.2 (-8.9, -3.6)	<0.0001	81.8 (80.2, 83.4)	76.9 (74.2, 79.6)	-4.9 (-7.4–2.3)	0.0003
HIV-infected not on antiretroviral therapy (N = 855)								
Fasting glucose (mmol/L)	6.6 (6.4, 6.6)	6.7 (6.4, 6.9)	0.1 (-0.2, 0.4)	0.46	6.6 (6.5, 6.6)	6.7 (6.4, 6.9)	0.1 (-0.1, 0.4)	0.29
Glucose at 30 min(mmol/L)	8.5 (8.4, 8.6)	8.4 (8.0, 8.7)	-0.1 (-0.5, 0.2)	0.43	8.5 (8.4, 8.6)	8.4 (8.1, 8.7)	-0.1 (-0.4, 0.2)	0.54
Glucose at 120 min (mmol/L)	8.5 (8.3, 8.7)	8.2 (7.8, 8.6)	-0.3 (-0.7, 0.2)	0.26	8.5 (8.3, 8.7)	8.3 (7.8, 8.7)	-0.2 (-0.7, 0.2)	0.24
HbA1c (%)	5.9 (5.8, 5.9)	5.8 (5.6, 6.0)	-0.1 (-0.3, 0.2)	0.68	5.8 (5.8, 5.9)	5.8 (5.6, 6.0)	-0.03(-0.3, 0.2)	0.76
Fat mass (kg)	11.4 (10.9, 11.9)	10.0 (7.9, 11.9)	-1.4 (-3.5, 0.6)	0.18	10.7 (9.3, 11.6)	9.6 (7.3, 11.8)	-1.1 (-3.2, 0.9)	0.28
Waist circumference (cm)	77.0 (76.4, 77.9)	75.7 (73.4, 78.0)	-1.3 (-3.8, 1.1)	0.28	76.7 (75.7, 77.8)	75.9 (73.4, 78.4)	-0.8 (-3.2, 1.6)	0.49
HIV infected on antiretroviral therapy (N = 294)								
Fasting glucose (mmol/L)	6.7(6.6, 6.9)	6.6 (6.4, 6.8)	-0.1 (-0.4, 0.1)	0.24	6.7 (6.6, 6.9)	6.6 (6.4, 6.8)	-0.1 (-0.4, 0.2)	0.43
Glucose at 30 min(mmol/L)	8.6 (8.4, 8.8)	8.3 (7.7, 8.8)	-0.3 (-0.9, 0.3)	0.27	8.6 (8.4, 8.8)	8.3 (7.8, 8.9)	-0.3 (-0.9, 0.3)	0.35
Glucose at 120 min (mmol/L)	8.0 (7.7, 8.3)	7.9 (7.6, 8.2)	-0.1 (-0.5, 0.3)	0.66	8.0 (7.7, 8.3)	7.9 (7.6, 8.2)	0.04 (-0.4, 0.4)	0.83
HbA1c (%)	5.5 (5.4, 5.7)	5.4 (5.2, 5.7)	-0.1 (-0.4, 0.2)	0.44	5.5 (5.4, 5.7)	5.5 (5.3, 5.8)	-0.02 (-0.3, 0.3)	0.87
Fat mass (kg)	11.0 (10.2, 11.9)	10.5 (8.5, 12.6)	-0.5 (-2.7, 1.7)	0.66	9.3 (8.1, 10.6)	9.5 (7.3, 11.8)	0.2 (-1.9, 2.4)	0.85
Waist circumference (cm)	78.4 (77.2, 79.5)	78.5 (75.4, 81.6)	0.1 (-0.3, 3.5)	0.91	75.8 (74.1, 77.5)	76.8 (73.7, 80.1)	1.0 (-2.1, 4.2)	0.52

^a^Adjusted for age, sex, C-Reactive Protein, malaria infection, tuberculosis, body mass index, and physical activity in estimating association with glucose (fasting, 30 and 120 min) and HbA1c

^b^Adjusted for age, sex, C-Reactive Protein, malaria infection, tuberculosis, smoking, alcohol drinking and physical activity in estimating association with fat mass and waist circumference.

## Discussion

In this study, we had hypothesized that helminth infection in HIV-uninfected participants would be associated with better insulin sensitivity and β-cell function whereas helminth-HIV co-infection would increase the risk of insulin resistance and β-cell function as result of severe immune activation and chronic inflammation [39, 40]. In agreement with our hypothesis, this analysis found that *Schistosoma* infection was associated with higher level of insulin secretion among HIV-uninfected participants. In addition, among participants with *Schistosoma* or geohelminth infection there was reduced insulin secretion among HIV-infected participants not yet on ART. Contrary to our hypothesis, among HIV-uninfected participants geohelminths were associated with reduced insulinogenic index and among HIV-infected participants not on ART *Schistosoma* infection was associated with reduced insulin resistance. Overall, these metabolic changes were not associated with corresponding changes in serum glucose levels or HbA1c.

### Beneficial effects of *Schistosoma* and geohelminth infections

Several studies have reported associations of *Schistosoma* and geohelminth infections with metabolic diseases. A recent study in Uganda found no association of helminths with insulin resistance or glucose [41], but in Ethiopia investigators found in a small study that *S*. *mansoni* infection was associated with reduced risk of impaired fasting glucose, but not with insulin secretion [42]. Outside SSA, onene Chinese study by Yuhong and colleagues found that history of previous *S*. *mansoni* infection was associated with reduced risk of diabetes and better metabolic profile among adults aged >60 years [43]. Similarly, Wiria and colleagues found that helminths were associated with modest improvement in insulin sensitivity not accounted by body mass index reduction alone [44]. Most previous studies investigated the role of helminths on insulin resistance and not both insulin resistance and β-cell function. Therefore, our study adds novel data on this subject. We think at least two mechanisms could explain the beneficial effect of schistosomes on β-cell function among HIV-uninfected participants found in our study. First, *Schistosoma* infection could have reduced the negative effects of pro-inflammatory cytokines, including interleukin-1beta (IL-1β), tumour necrosis factor-α (TNF-α) and gamma-interferon (γ-IFN) on islet β cells [45] by shifting Th1 to Th2 immune response [4, 5]. In animal studies it has been shown that extracts of soluble *S*. *mansoni* worm or eggs antigens induced secretion of anti-inflammatory cytokines including IL-10, IL-4 and IL-5 from T cells and subsequently prevented development of type 1 diabetes in non-obese mice [46]. Similarly, in a group of mice with diabetes induced with streptozotocin (pancreatic islets β-cell toxin), those infected with *Schistosoma* mansoni had more pancreatic β-cells mass and less focal degeneration as well as lower glucose level in comparison to those without *Schistosoma* infection [47]. Second, by switching Th1 to Th2 immunomodulation profile, *Schistosoma* infection could have reduced white adipose tissue inflammation, and subsequently leading to reduced insulin resistance [48]. The reduced insulin resistance would have resulted in reduced β-cells glucotoxicity [49] contributing to improved β-cell function. However, we found that *Schistosoma* infection was not associated with reduced insulin resistance. Although this could have been due to the fact that the predictive ability of HOMA-IR on insulin resistance was only modest in this population [50].

Although geohelminth infections were not associated with improved β-cell function, possibly due to lack of strong immune-modulatory effects [26], they were associated with lower total fat mass as well as reduced waist circumference independent of physical activity. This could have been due to loss of appetite associated with systemic or intestinal infections, but this is unlikely since it was independent of systemic inflammation (measured by CRP) and was observed in HIV-uninfected participants but not among HIV-infected participants, the population group at a higher risk of experiencing loss of appetite. So these changes were mostly likely a reflection of body weight reduction which is characteristic of helminth infections [48]. In mice studies, administration of *Schistosoma* egg antigens were associated with reduced risk of obesity [48, 51]. Additionally, Wiria and colleagues found parasite intensity was negatively associated with body weight [44] and a Chinese study observed previous schistosome intensity was associated with current weight [43]. Reduction of abdominal fat is known to reduce the risk of diabetes, although this was not evident in the current study. This may have been because the observed loss in waist circumference was only modest.

### Association of *Schistosoma* and geohelminth infections among HIV-infected patients

In this analysis we found that among HIV-infected patients, geohelminth and *Schistosoma* infections were associated with reduced insulin secretion, although this was not accompanied by corresponding higher glucose or HbA1c level. This is in accordance with studies that had shown that schistosomiasis could worsen HIV progression [52] and that immunological shift from Th1 to Th2, leaves the body unarmed to combat viral and bacterial infections which could lead to severe infections and subsequently to insulin resistance [39, 40]. Insulin resistance resulting in hyperglycaemia could have led to reduced β-cell function secondary to glucotoxicity [17].

However, we found no effect on insulin resistance despite the fact that insulin resistance is commonly associated with systemic inflammation among HIV-infected patients. It is therefore possible that the negative effect on β-cell function could be explained by other mechanisms including direct deleterious effects of pro-inflammatory cytokines on β-cells. Further research to understand mechanisms underlying development of helminth-associated β-cell function and possibly insulin resistance would help in developing strategies to prevent or manage diabetes in these populations.

### Implications of results

Although *Schistosoma* and geohelminth infections should be prevented and treated, such measures could remove protection against diabetes and other metabolic diseases among HIV-uninfected populations. Although this is not a justification to withdraw prevention or treatment modalities, these measures should be implemented alongside other strategies to reduce risk of metabolic diseases including promotion of physical activity, weight reduction, consumption of healthy diet as well as avoiding excessive alcohol intake. Such non-communicable diseases (NCDs) prevention strategies are important to HIV-infected individuals particularly those not yet on ART since although reduced β-cell function was not related to overt hyperglycaemia these patients could quickly develop hyperglycaemia if they harbor other risk factors. Similarly, it is important to encourage early initiation as well as lifelong adherence of ART to reduce risk of β-cell function associated with *Schistosoma* and geohelminth infections. This is important because despite the roll out of the universal test-and treat policy which encourages HIV testing and immediate uptake of ART, many HIV patients do not start ART in timely fashion and, of those starting, up to 50% are lost to follow-up or become non-adherent within 3 years of starting treatment [53] thus increasing their diabetes risk. Finally, in view of these results, trials testing effects of *Schistosoma* or geohelminths derived antigens on risk of metabolic diseases should be encouraged to help develop interventions for the prevention of diabetes and other NCDs.

### Strengths and weaknesses

The strength of this study is that it included both HIV-infected and un-infected to assess the role of *Schistosoma* and geohelminth-HIV coinfection on β-cell function and insulin resistance; thus results can be generalized to wider populations in SSA where these helminths and HIV have high prevalence and overlap widely. In addition, the prevalence of *Schistosoma* and geohelminth infections was based on stool/urine examination and not symptom-based algorithms thus reducing potential for misclassification bias. However, in the assessment of helminths we only collected one day stool samples and diagnostic methods used have lower sensitivity compared to molecular and immunodiagnostic methods [54, 55], thus we may have underestimated the helminth prevalence. Also we did ask participants to refrain from physical activity during fasting because we knew most of our participants would walk or catch a public transport to our research clinic, but we expected this to be short with minimal effect in lowering glucose level. This study was cross-sectional and therefore causality cannot be confirmed. Although we controlled for potential confounders, we cannot rule out that there remained residual confounding. Finally, the sample size for participants on ART was the smallest, despite this being an important group given that most HIV patients should be on ART. Future larger studies should assess the association of helminth infections on β-cell function and insulin resistance among patients on ART.

## Conclusion

In conclusion, in this high HIV burden setting, we found that *Schistosoma* infection was associated with better β-cell function among HIV-uninfected participants whereas *Schistosoma* and geohelminth infections were associated with reduced β-cell function among HIV-infected patients not yet on ART. Future larger studies are needed to confirm results that helminths are not associated with β-cell function or insulin resistance among patients on ART.

## Supporting information

S1 Table Markers of β-cell function and insulin resistance. (DOCX)

S2 Table Background characteristics of CICADA participants included and those not included in the analysis. (DOCX)

S3 Table Analysis of association of *Schistosoma* infection with β-cell function and insulin resistance. (DOCX)

S4 Table Analysis of association of geohelminth infection with β-cell function and insulin resistance. (DOCX)

S5 Table Analysis of association of *Schistosoma* infection with glucose, HbA1c, fat mass, and waist circumference. (DOCX)

S6 Table Analysis of association of geohelminth infection with glucose, HbA1c, fat mass, and waist circumference. (DOCX)

S7 Table Prevalence of schistosomiasis by HIV treatment status. (DOCX)

S8 Table Prevalence of geohelminths by treatment HIV status. (DOCX)
