# Supplementary material for: The association of Schistosoma and geohelminth infections with β-cell function and insulin resistance among HIV-infected and HIV-uninfected adults: A cross-sectional study in Tanzania
Source: PLoS One. 2022 Jan 25;17(1):e0262860. doi: 10.1371/journal.pone.0262860 (PMC8789133; doi:10.1371/journal.pone.0262860)
Supplement: S1 Table — (DOCX) [file pone.0262860.s001.docx]

| S1 Table: Markers of β-cell function and insulin resistance | | | |
| --- | --- | --- | --- |
| Marker | Definition/formula | Units | References |
| β-cell function |  |  |  |
| Fasting insulin | - | mU/L | - |
| Insulin at 30 min | - | mU/L | - |
| Insulin at 120 min | - | mU/L | - |
| Insulinogenic index | Change in insulin over change in glucose in first 30 minutes following OGTT. | (mU/L/mg/dL) | (45) |
| HOMA-β cell function | (20* Fasting blood insulin (FBI)/(Fasting plasma glucose (FPG)-3.5) | (mU/L/mmol/L) | (25) |
| Overall insulin release index | Ratio of AUC of insulin to AUC of glucose from 0 to 120 minutes of OGTT | (pmol/L/mmol/L) | (23) |
| Insulin resistance(IR) |  |  |  |
| HOMA-IR | (FBI *FPG)/22.5 | (mU/L/mmol/L) | (25) |
| AUC, area under the curve; HOMA-β, Homeostatic model assessment-β; HOMA-IR, HOMA-Insulin Resistance; OGTT, Oral glucose tolerance test; | | | |
