# Supplementary material for: The association of Schistosoma and geohelminth infections with β-cell function and insulin resistance among HIV-infected and HIV-uninfected adults: A cross-sectional study in Tanzania
Source: PLoS One. 2022 Jan 25;17(1):e0262860. doi: 10.1371/journal.pone.0262860 (PMC8789133; doi:10.1371/journal.pone.0262860)
Supplement: S2 Table — (DOCX) [file pone.0262860.s002.docx]

| S2 Table. Background characteristics of CICADA participants included and those not included in the analysis | | | |
| --- | --- | --- | --- |
|  | Participants included (N=718) | Participants not included (N=229) | *P* |
| Age (years), mean (SD) | 40.7 (11.9) | 40.4 (12.4) | 0.72 |
| Female sex, n(%) | 997 (58.1) | 160 (69.7) | 0.01 |
| Social economic status, n(%)^a, b^ |  |  |  |
| Lower | 567 (33.1) | 81 (35.7) | 0.69 |
| Medium | 576 (33.6) | 71 (31.3) |  |
| Higher | 572 (33.4) | 75 (33.0) |  |
| Body mass index (kg), mean (SD) | 23.8 (4.9) | 22.5 (3.2) |  |
| Physically activity (MET min per week ), mean (SD) | 9106 (7320)^c^ | 8963.0 (6646.4)^b^ | 0.78 |
| Smoking status, n(%)^a,b^ |  |  |  |
| Never | 1292 (75.3) | 182 (80.2) | 0.16 |
| Past | 255 (14.9) | 31 (13.7) |  |
| Current | 168 (8.5) | 14 (6.1) |  |
| Ever taken alcohol, n (%) ^a,b^ | 1236 (72.1) | 153 (67.4) | 0.14 |
| C-Reactive Protein (mg/L)^i^ | 2.8 (2.6, 3.0)^d^ | 2.8 (23, 3.5)^d^ | 0.95 |
| ^a^3participants missing in included group ^b^2 participants missing in the excluded group  ^c^4participants missing ^d^14participants missing | | | |
