# Supplementary material for: The association of Schistosoma and geohelminth infections with β-cell function and insulin resistance among HIV-infected and HIV-uninfected adults: A cross-sectional study in Tanzania
Source: PLoS One. 2022 Jan 25;17(1):e0262860. doi: 10.1371/journal.pone.0262860 (PMC8789133; doi:10.1371/journal.pone.0262860)
Supplement: S3 Table — (DOCX) [file pone.0262860.s003.docx]

| S3 Table. Analysis of association of *Schistosoma* infection with β-cell function and insulin resistance | | | | | | | | |  |
| --- | --- | --- | --- | --- | --- | --- | --- | --- | --- |
|  | Age and sex adjusted model | | | | Fully adjusted model^a^ | | | | *P^a^* |
|  | Marginal means (95% CI) | | | *P* | Marginal means (95% CI) | | | *P* |  |
|  | *Schistosoma*-uninfected | *Schistosoma*-infected | Difference |  | *Schistosoma*-uninfected | *Schistosoma* infected | Difference |  |  |
| Insulin level during OGTT |  |  |  |  |  |  |  |  |  |
| Fasting insulin (mU/L) | 6.6 (6.2, 6.9) | 5.7 (5.1, 6.3) | -0.9 (-1.5, -0.2) | 0.01 | 6.3 (6.1, 6.6) | 5.9 (5.4, 6.4) | -0.4 (-0.9, 0.1) | 0.12 | 0.06 |
| Insulin at 30 min (mU/L) | 50.8 (48.7, 52.9) | 55.5 (47.9, 63.0) | 4.7 (-3.1, 12.5) | 0.24 | 51.0 (49.0, 53.0) | 58.3 (50.6, 65.9) | 7.2 (-0.7, 15.1) | 0.07 | 0.02 |
| Insulin at 120 min (mU/L) | 47.1 (45.2, 49.1) | 41.3 (35.1, 47.6) | -5.8 (-12.4, 0.7) | 0.08 | 47.7 (45.8, 49.5) | 42.0 (36.8, 47.3) | -5.6 (-11.1, -0.1) | 0.04 | 0.92 |
| Markers of β-cell function |  |  |  |  |  |  |  |  |  |
| HOMA-β (mU/L)/(mmol/L) | 46.2 (44.1, 48.2) | 43.7 (37.4, 49.3) | -2.5 (-9.1, 4.1) | 0.46 | 46.1 (44.1, 48.0) | 46.6 (40.6, 52.5) | 0.5 (-5.7, 6.7) | 0.88 | 0.60 |
| Insulinogenic index (mU/L)/(mg/dL) | 1.5 (1.4, 1.7) | 2.2 (1.4, 3.0) | 0.7 (-0.1, 1.5) | 0.11 | 1.5 (1.4, 1.7) | 2.3 (1.5, 3.2) | 0.8 (-0.005, 1.7) | 0.05 | 0.45 |
| Overall insulin release index (pmol/L/mmol/L) | 38.0 (36.7, 39.4) | 39.4 (34.6, 43.4) | 1.0 (-3.7, 5.6) | 0.68 | 37.9 (36.7, 39.2) | 40.4 (36.2, 44.6) | 2.5 (-1.8, 6.8) | 0.26 | 0.01 |
| Marker of insulin resistance |  |  |  |  |  |  |  |  |  |
| HOMA-IR (mU/L)/(mmol/L) | 2.0 (1.9, 2.1) | 1.7 (1.4, 2.0) | -0.3 (-0.6, 0.04) | 0.09 | 1.9 (1.9, 2.0) | 1.8 (1.6, 2.0) | -0.1 (-0.3, 0.1) | 0.26 | 0.17 |
| HOMA-β, Homeostatic model assessment-β; HOMA-IR, HOMA-Insulin Resistance; OGTT, oral glucose tolerance test. ^a^Adjusted for age, sex, C-Reactive Protein, body mass index, and physical activity. *^a^P*, test for interaction with HIV treatment status (HIV-uninfected, HIV-infected not on antiretroviral therapy (ART) and HIV infected on ART) | | | | | | | | | |
