# Supplementary material for: The association of Schistosoma and geohelminth infections with β-cell function and insulin resistance among HIV-infected and HIV-uninfected adults: A cross-sectional study in Tanzania
Source: PLoS One. 2022 Jan 25;17(1):e0262860. doi: 10.1371/journal.pone.0262860 (PMC8789133; doi:10.1371/journal.pone.0262860)
Supplement: S4 Table — (DOCX) [file pone.0262860.s004.docx]

| S4 Table. Analysis of association of geohelminth infection with β-cell function and insulin resistance | | | | | | | | | |
| --- | --- | --- | --- | --- | --- | --- | --- | --- | --- |
|  | Age and sex adjusted model | | | | Fully adjusted model^a^ | | | | *P^a^* |
|  | Marginal means (95% CI) | | | *P* | Marginal means (95% CI) | | | *P* |  |
|  | Geohelminth-uninfected | Geohelminth- infected | Difference |  | Geohelminth-uninfected | Geohelminth-infected | Difference |  |  |
| Insulin level during OGTT |  |  |  |  |  |  |  |  |  |
| Fasting insulin (mU/L) | 6.6 (6.2, 6.9) | 5.7 (5.0, 6.3) | -0.9 (-1.6, -0.2) | 0.01 | 6.3 (6.1, 6.6) | 6.0 (5.4, 6.6) | -0.3 (-0.9, 0.3) | 0.29 | 0.47 |
| Insulin at 30 min (mU/L) | 50.8 (48.7, 52.9) | 49.0 (42.7, 55.3) | -1.8 (-8.5, 4.8) | 0.58 | 51.0 (49.0, 52.9) | 52.3 (46.3, 58.4) | 1.4.0(-4.9, 7.6) | 0.67 | 0.46 |
| Insulin at 120 min (mU/L) | 47.2 (45.3, 49.1) | 41.5 (34.8, 48.2) | -5.7 (-12.7, 1.22) | 0.11 | 47.6 (45.8, 49.5) | 44.0 (38.5, 49.5) | -3.6 (-9.3, 2.1) | 0.21 | 0.44 |
| Markers of β-cell function |  |  |  |  |  |  |  |  |  |
| HOMA-β (mU/L)/(mmol/L) | 46.1 (44.1, 48.2) | 38.9 (33.3, 44.6) | -7.2 (-13.2, -1.2) | 0.02 | 46.0 (44.1, 47.9) | 42.1 (36.7, 47.5) | -3.9 (-9.5, 1.7) | 0.17 | 0.23 |
| Insulinogenic index (mU/L)/(mg/dL) | 1.5 (1.3, 1.7) | 1.4 (0.9, 1.9) | -0.1 (-0.7,0.4) | 0.64 | 1.5 (1.3, 1.7) | 1.5 (0.9, 2.1) | 0.01 (-0.5, 0.6) | 0.97 | 0.02 |
| Overall insulin release index (pmol/L/mmol/L) | 38.1 (36.7, 39.4) | 35.5 (31.4, 39.6) | -2.6 (-6.9, 1.7) | 0.24 | 37.9 (36.7, 39.1) | 37.3 (33.5, 44.1) | -0.6 (-4.5, 3.3) | 0.76 | 0.08 |
| Marker of insulin resistance |  |  |  |  |  |  |  |  |  |
| HOMA-IR (mU/L)/(mmol/L) | 1.9 (1.8, 2.1) | 1.7 (1.4, 2.0) | -0.2 (-0.6, 0.05) | 0.11 | 1.9 (1.8, 2.0) | 1.9 (1.7, 2.1) | -0.04 (-0.3,0.2) | 0.72 | 0.53 |
| HOMA-β, Homeostatic model assessment-β; HOMA-IR, HOMA-Insulin Resistance; OGTT, oral glucose tolerance test.  ^a^Adjusted for age, sex, C-Reactive Protein, body mass index , and physical activity. *^a^P*, test for interaction with HIV treatment status (HIV-uninfected, HIV-infected not on antiretroviral therapy (ART) and HIV infected on ART) | | | | | | | | | |
