# Supplementary material for: The association of Schistosoma and geohelminth infections with β-cell function and insulin resistance among HIV-infected and HIV-uninfected adults: A cross-sectional study in Tanzania
Source: PLoS One. 2022 Jan 25;17(1):e0262860. doi: 10.1371/journal.pone.0262860 (PMC8789133; doi:10.1371/journal.pone.0262860)
Supplement: S5 Table — (DOCX) [file pone.0262860.s005.docx]

| S5 Table. Analysis of association of *Schistosoma* infection with glucose, HbA1c, fat mass, and waist circumference | | | | | | | | | |
| --- | --- | --- | --- | --- | --- | --- | --- | --- | --- |
|  | Age and sex adjusted model | | | | Fully adjusted model^a, b^ | | | | *P^a^* |
|  | Marginal means (95% CI) | | | *P* | Marginal means (95% CI) | | | *P* |  |
|  | *Schistosoma*-uninfected | *Schistosoma*- infected | Difference |  | *Schistosoma*-uninfected | *Schistosoma*-infected | Difference |  |  |
| HIV-uninfected participants (N=569) |  | | | |  | | | |  |
| Fasting glucose (mmol/L) | 6.6 (6.5, 6.7) | 6.6 (6.3, 6.8) | -0.03 (-0.2, 0.2) | 0.77 | 6.6 (6.5, 6.7) | 6.6 (6.3, 6.8) | -0.03 (-0.3, 0.2) | 0.77 | 0.94 |
| Glucose at 30 min(mmol/L) | 8.5 (8.4, 8.6) | 8.5 (8.1, 8.8) | -0.02 (-0.3, 0.3) | 0.90 | 8.5 (8.4, 8.6) | 8.5 (8.1, 8.8) | -0.03 (-0.4, 0.3) | 0.84 | 0.92 |
| Glucose at 120 min (mmol/L) | 8.3 (8.1, 8.4) | 8.2 (7.7, 8.7) | -0.09 (-0.5, 0.4) | 0.70 | 8.3 (8.1, 8.4) | 8.2 (7.7, 8.6) | -0.09 (-0.6, 0.4) | 0.70 | 0.85 |
| HbA1c (%) | 5.7 (5.6, 5.7) | 5.7 (5.5, 5.9) | -0.01 (-0.2, 0.1) | 0.89 | 5.7 (5.6, 5.7) | 5.7 (5.5, 5.9) | -0.01 (-0.2, 0.2) | 0.93 | 0.43 |
| Fat mass (kg) | 12.9 (12.5, 13.4) | 12.0 (10.8, 13.3) | -0.9 (-2.2, 0.3) | 0.15 | 11.6 (11.1, 12.3) | 11.1 (9.9, 12.4) | -0.5 (-1.7, 0.7) | 0.43 | 0.52 |
| Waist circumference (cm) | 79.5 (78.9, 80.0) | 78.4 (76.9, 79.9) | -1.1 (-2.6, 0.5) | 0.18 | 78.1 (77.3, 78.9) | 77.4 (75.9, 78.9) | -0.6 (-2.1, 0.9) | 0.41 | 0.77 |
| ^a^Adjusted for age, sex, C-Reactive Protein, body mass index, and physical activity in estimating association with glucose (fasting, 30 and 120 min) and HbA1c  ^b^Adjusted for age, sex, C-Reactive Protein, smoking, alcohol drinking and physical activity in estimating association with fat mass and waist circumference.  *^a^P*, test for interaction with HIV treatment status (HIV-uninfected, HIV-infected not on antiretroviral therapy (ART) and HIV infected on ART) | | | | | | | | | |
