# Supplementary material for: The association of Schistosoma and geohelminth infections with β-cell function and insulin resistance among HIV-infected and HIV-uninfected adults: A cross-sectional study in Tanzania
Source: PLoS One. 2022 Jan 25;17(1):e0262860. doi: 10.1371/journal.pone.0262860 (PMC8789133; doi:10.1371/journal.pone.0262860)
Supplement: S6 Table — (DOCX) [file pone.0262860.s006.docx]

| S6 Table. Analysis of association of geohelminth infection with glucose, HbA1c, fat mass, and waist circumference | | | | | | | | | | | |
| --- | --- | --- | --- | --- | --- | --- | --- | --- | --- | --- | --- |
|  | Age and sex adjusted model | | | | | Fully adjusted model^a, b^ | | | | | *P^a^* |
|  | Marginal means (95% CI) | | | | *P* | Marginal means (95% CI) | | | | *P* |  |
|  | geohelminth-uninfected | geohelminth- infected | | Difference |  | geohelminth-uninfected | geohelminth-infected | | Difference |  |  |
| Fasting glucose (mmol/L) | 6.6 (6.5, 6.7) | | 6.7 (6.4, 6.8) | 0.06 (-0.1, 0.2) | 0.49 | 6.6 (6.5, 6.7) | | 6.7 (6.5, 6.9) | 0.1 (-0.05, 0.3) | 0.19 | 0.24 |
| Glucose at 30 min(mmol/L) | 8.5 (8.4, 8.6) | | 8.4 (8.1, 8.6) | -0.1 (-0.4, 0.1) | 0.31 | 8.5 (8.4, 8.6) | | 8.4 (8.2, 8.7) | -0.06 (-0.3, 0.2) | 0.58 | 0.58 |
| Glucose at 120 min (mmol/L) | 8.3 (8.1, 8.4) | | 8.0 (7.7, 8.3) | -0.3 (-0.6, 0.01) | 0.06 | 8.3 (8.1, 8.4) | | 8.0 (7.8, 8.3) | -0.2 (-0.5, 0.05) | 0.11 | 0.11 |
| HbA1c (%) | 5.7 (5.6, 5.7) | | 5.5 (5.4, 5.6) | -0.2 (-0.3, -0.03) | 0.02 | 5.6 (5.6, 5.7) | | 5.5 (5.4, 5.6) | -0.1 (-0.2, 0.01) | 0.09 | 0.22 |
| Fat mass (kg) | 13.0(12.5, 13.3) | | 10.5 (9.3, 11.5) | -2.5 (-3.7, -1.3) | <0.0001 | 11.7 (11.2, 12.4) | | 9.6 (8.4, 10.8) | -2.1 (-3.3, -0.9) | 0.004 | 0.005 |
| Waist circumference (cm) | 79.5 (78.9, 80.1) | | 76.9 (75.4, 78.4) | -2.6 (-4.1, -1.0) | 0.001 | 78.2 (77.4, 79.0) | | 75.9 (74.4, 77.5) | -2.3 (-3.7, -0.7) | 0.004 | 0.003 |
| ^a^Adjusted for age, sex, C-Reactive Protein, body mass index, and physical activity in estimating association with glucose (fasting, 30 and 120 min) and HbA1c  ^b^Adjusted for age, sex, C-Reactive Protein, smoking, alcohol drinking and physical activity in estimating association with fat mass and waist circumference.  *^a^P*, test for interaction with HIV treatment status (HIV-uninfected, HIV-infected not on antiretroviral therapy (ART) and HIV infected on ART) | | | | | | | | | | | |
