# Supplementary material for: The association of Schistosoma and geohelminth infections with β-cell function and insulin resistance among HIV-infected and HIV-uninfected adults: A cross-sectional study in Tanzania
Source: PLoS One. 2022 Jan 25;17(1):e0262860. doi: 10.1371/journal.pone.0262860 (PMC8789133; doi:10.1371/journal.pone.0262860)
Supplement: S7 Table — (DOCX) [file pone.0262860.s007.docx]

| S7 Table. Prevalence of schistosomiasis by HIV treatment status | | | |
| --- | --- | --- | --- |
|  | HIV-uninfected participants (N=569) | HIV-infected not on ART (N=855) | HIV-infected on ART (N=294) |
| *S. Haematobium, n(%)* | 1 (0.2) | 0 | 0 |
| *S. Mansoni*, n (%) | 50 (8.8) | 70 (8.2) | 18 (6.1) |
| Total schistosoma infection, n (%) | 51 (8.9) | 70 (8.2) | 18 (6.1) |

ART, Antiretroviral therapy
