# Supplementary material for: The association of Schistosoma and geohelminth infections with β-cell function and insulin resistance among HIV-infected and HIV-uninfected adults: A cross-sectional study in Tanzania
Source: PLoS One. 2022 Jan 25;17(1):e0262860. doi: 10.1371/journal.pone.0262860 (PMC8789133; doi:10.1371/journal.pone.0262860)
Supplement: S8 Table — (DOCX) [file pone.0262860.s008.docx]

| S8 Table. Prevalence of geohelminths by HIV treatment status | | | |
| --- | --- | --- | --- |
|  | HIV-uninfected participants (N=569) | HIV-infected not on ART (N=855) | HIV-infected on ART (N=294) |
| *Ascaris lumbricoides, n(%)* | 3 (0.5) | 7 (0.8) | 9 (3.0) |
| Hookworms, n (%) | 42 (7.4) | 47 (5.5) | 19 (6.5) |
| *T. trichiura*, n(%) | 4 (0.7) | 5 (0.6) | 2 (0.7) |
| *S. stercoralis*, n(%) | 4 (0.7) | 9 (1.1) | 6 (2.0) |
| Helminth infection with Schistosomiasis co-infection, n(%)^a^ | 4 (0.7) | 8 (0.9) | 3 (1.0) |
| Total geohelminth infections only, n(%) | 48 (8.4) | 57 (6.7) | 31 (10.5) |

^a^Regarded as schistosomiasis in analysis ART, Antiretroviral therapy
